# Thermodynamic and microstructural study of Ti_2_AlNb oxides at 800 °C

**DOI:** 10.1038/s41598-018-31196-w

**Published:** 2018-08-24

**Authors:** J. M. Xiang, G. B. Mi, S. J. Qu, X. Huang, Z. Chen, A. H. Feng, J. Shen, D. L. Chen

**Affiliations:** 1 School of Materials Science and Engineering, Tongji University, Shanghai, 201804 P.R. China; 2 Aviation Key Laboratory of Science and Technology on Advanced Titanium Alloys, AECC Beijing Institute of Aeronautical Materials, Beijing, 100095 China; 3 Aerospace Hiwing (Harbin) Titanium Industrial Co., Ltd., Harbin, 150028 China; 4 Department of Mechanical and Industrial Engineering, Ryerson University, Toronto, Ontario M5B 2K3 Canada

## Abstract

The high-temperature structural applications of Ti_2_AlNb-based alloys, such as in jet engines and gas turbines, inevitably require oxidation resistance. The objective of this study is to seek fundamental insight into the oxidation behavior of a Ti_2_AlNb-based alloy via detailed microstructural characterization of oxide scale and scale/substrate interface after oxidation at 800 °C using X-ray diffraction (XRD), scanning electron microscopy (SEM), electron probe microanalysis (EPMA), and transmission electron microscopy (TEM). The oxide scale exhibits a complex multi-layered structure consisting of (Al,Nb)-rich mixed oxide layer (I)/mixed oxide layer (II)/oxygen-rich layer (III)/substrate from the outside to inside, where the substrate is mainly composed of B2 and O-Ti_2_AlNb phases. High-resolution TEM examinations along with high-angle annular dark-field (HAADF) imaging reveal: (1) the co-existence of two types (α and δ) of Al_2_O_3_ oxides in the outer scale, (2) the presence of metastable oxide products of TiO and Nb_2_O_5_, (3) an amorphous region near the scale/substrate interface including the formation of AlNb_2_, and (4) O-Ti_2_AlNb phase oxidized to form Nb_2_O_5_, TiO_2_ and Al_2_O_3_.

## Introduction

Ti_2_AlNb-based alloy, sometimes referred to as orthorhombic alloy^1,2^, is a class of highly promising lightweight high-temperature materials. This type of alloy is considered to partially substitute the high-density (*ρ* = 8~8.5 g/cm^3^) Ni-based superalloys in the aerospace industry due to its low density, high strength, superior plasticity, high fracture toughness and excellent creep resistance at elevated temperatures^3–9^. In such applications, the operating temperatures could go beyond 600–650 °C^10,11^, leading to severe oxidation of the alloy surface^12–14^. There are three potential approaches to improve high-temperature oxidation resistance: alloying^15–17^, pre-oxidation^18^, and coating^19–26^. For example, Wang *et al*.^27^ reported that a two-step voltage-controlled microarc oxidation (MAO) method can be used to produce ceramic coatings on a Ti_2_AlNb-based alloy. However, after a prolonged exposure to air at elevated temperatures, intermetallics exhibit oxygen-induced embrittlement characteristics such as low ductility and brittle fracture^16,28,29^. Thus, an understanding of high-temperature oxidation mechanisms is essential for improving the oxidation resistance of materials. In our previous study^30^, a Ti_2_AlNb-based alloy was observed to exhibit fairly good oxidation resistance below 750 °C. After reaching 800 °C, the oxidation resistance decreased dramatically. Thus, the oxidation behavior and mechanisms are investigated at a higher temperature of 800 °C in this study.

Mass transfer is known to be the essence of oxidation reaction. During the high-temperature oxidation of a Ti_2_AlNb-based alloy, O and N elements diffuse inward, whereas Al, Ti and Nb elements diffuse outward. Among the many elements that can improve the oxidation resistance, such as Al, Nb, Mo, Si, Zr, etc., Al and Nb are the most important elements^31^. While Al atoms and O atoms are able to generate a continuous and dense Al_2_O_3_ protective layer on the alloy surface and thus improve the oxidation resistance of alloys, this is not the case in Ti_2_AlNb-based alloys. The Gibbs free energy of Al_2_O_3_ and TiO_2_ is so similar that both oxides are produced almost simultaneously^32^. The addition of the element Nb can improve the oxidation resistance of the alloy: Nb substitutes for Ti in TiO_2_ as a cation with a valence of 5, while no Nb is present in Al_2_O_3_^33–37^. The doping of Nb in TiO_2_ grains reduces oxygen vacancy and Ti cations, which impedes the mass transfer in TiO_2_^34^. Lu *et al*.^35^ observed the substitution of Ti by Nb via high-resolution transmission electron microscopy (HRTEM) Z-contrast imaging, as represented by the Nb enrichment in TiO_2_ grains of the mixture layer. Vojtěch *et al*.^36^ studied the role of the addition of 2 at.% Nb to the eutectic TiAl-Ti_5_Si_3_ alloy, and reported that Nb markedly influences oxidation kinetics, with a six-fold decrease of oxidation rate.

Some fundamental aspects of oxidation behavior, such as weight gain, scale morphology, and structure, have been investigated^12,21,38–41^. Leyens and Gedanitz^12^ studied the mass gain and oxidation rate of a Ti-22Al-25Nb alloy in air between 650 °C and 800 °C, and reported a fairly good oxidation resistance at 650 °C up to 4000 hours and at 700 °C up to 500 hours, whereas at 800 °C “breakaway” oxidation occurred after about 100 hours. Wang *et al*.^21^ observed layers of TiO_2_ and a small amount of AlNbO_4_ with needle-like TiO_2_ crystals present all over the surface. Ralison *et al*.^38,39^ reported a multi-layered scale $({{\rm{TiO}}}_{2}+\frac{1}{2}{{\rm{AlNbO}}}_{4}/({\rm{eventually}}\,{\rm{Ta}},\,{\rm{Mo}})-{\rm{rich}}\,{{\rm{AlNbO}}}_{4})$ along with an oxygen-affected zone in a Ti-27Al-15Nb alloy at 800 °C in air, and Al_2_O_3_/(TiO_2_ + AlNbO_4_)/Ta-rich Al_2_O_3_/oxygen-affected zone in a Ti-27Al-10Nb alloy at the same temperature of 800 °C. Some cracks were present in the multi-layered scale. Zheng *et al*.^40^ studied the oxidation behavior of a Ti-22Al-25Nb alloy at 800 °C for 300 hours and observed the formation of a mixed oxide scale on the alloy surface, which was predominantly composed of TiO_2_, AlNbO_4_, and Nb_2_O_5_.

These studies revealed a complex scale structure containing oxidation products of Al_2_O_3_, TiO_2_, Nb_2_O_5_, AlNbO_4_, etc., with an outer layer consisting mainly of TiO_2_. When the content of the element Nb is high enough, Nb_2_O_5_ or AlNbO_4_ would be present, however, they are prone to spall-off and are unfavorable to the oxidation resistance. As for the structure of inner oxide layer, Małecka^41^ observed that it consists of an Al-rich layer and Ti, Al (Nb, Mo, V)-rich zone. Li *et al*.^42^ reported that it has such a structure: TiO_2_-rich layer/AlNbO_4_-rich layer/TiO_2_-rich layer/AlNbO_4_-rich layer/oxygen and a nitride-enriched zone. Leyens^14^ reported that when the temperature is above 900 °C there exists a nitrogen-enriched layer underneath the oxide scale, i.e., a nitride-containing layer. However, the questions remain as to how the oxide scale containing various oxides is formed; in what form (crystalline or amorphous) the substance/scale interface region would be; and if different types of Al_2_O_3_ can be co-existent in the oxidation of Ti_2_AlNb-based alloys. The objective of the present study is to address these questions via detailed microstructrual examinations using different advanced techniques along with thermodynamic calculations.

## Materials and Methods

The selected material is as-cast Ti_2_AlNb alloy with a nominal composition of Ti-22Al-20Nb-2V-1Mo-0.25Si (in at.%). The alloy ingot was cut into small plates with a size of 8 × 8 × 3 mm by electro-discharge machining. The surface of the samples was ground with sandpaper from grit #400 to #1200, ultrasonically cleaned in acetone for 15 mins. The dimensions were measured using a Vernier caliper and the samples were weighted using an analytical balance with an accuracy of 0.00001 g. During the isothermal oxidation in air at 800 °C, the samples were taken out of the furnace at intervals of 1, 3, 6, 12, 24, 36, 50, 62, 74, 86, and 100 h, cooled to room temperature and weighed. It should be noted that the Ti_2_AlNb-based alloy was observed to exhibit a fairly good oxidation resistance below 750 °C, while its oxidation resistance decreased considerably above 800 °C. Thus, the oxidation behavior and mechanism are studied in detail at 800 °C in the present investigation. The time of oxidation experiments was selected according to the standard HB5258-2000 of aerospace sector in China, where a duration of 100 hours is suggested to be sufficient. Also, if the oxidation time was too long, the oxide layer would peel off, causing difficulties for the study of the oxide scale.

A stereoscope was used to observe the grain sizes. XRD (Rigaku D/Max-2550) with a Cu K_α_ radiation (λ = 1.5418 Å) was used to identify the phases in the oxide scale at 50 kV and 200 mA with a diffraction angle (2θ) from 10° to 100° at a step size of 0.02° and 1 s in each step. SEM (Nova Nano SEM 450) was used to observe the surface morphology and cross-sectional structure of the oxide layer. For the observation of the oxide cross-section, it was necessary to mount the sample with resin, use sandpapers from grit #400 to #1200 to grind, and then diamond paste to polish the sample to a smooth mirror surface. Since the oxide is an insulator, it is necessary to perform carbon deposition on the polished surface before SEM observations. Elemental distribution in different regions on the cross-section of the oxide scale was characterized by using an electron probe microanalyzer (EPMA, Shimadzu 1720) with a resolution of 1 μm and secondary-electron image resolution of 6 nm using a beam current of 10 nA. TEM (FEI TECNAI G2 S-TWIN F20) was used to examine the structures of the oxide scale as well as the scale/substrate interface. To locate the scale/substrate interface more precisely TEM samples were prepared via the cutting of a focused ion beam (FIB, FEI, Helios nanolab 600). The dimension of FIB-TEM samples is: 5 μm in length, 4 μm in width, ~35 nm in thickness for the scale, and ~65 nm in thickness for the scale/substrate interface.

## Results

### Microstructures of as-cast Ti_2_AlNb alloy

**Figure 1:**
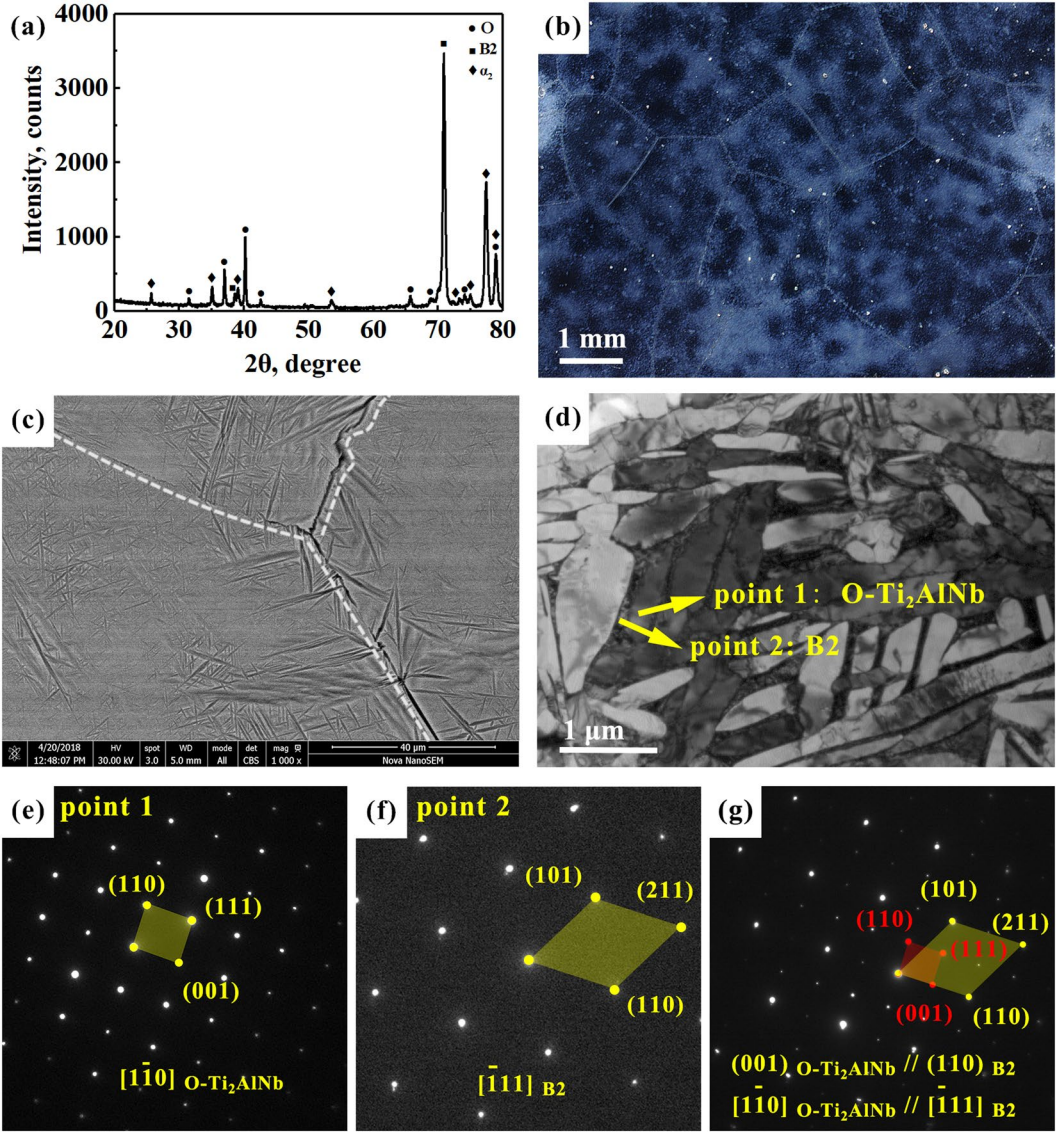
X-ray diffraction pattern (**a**) and microstructure (**b**–**g**) of as-cast Ti_2_AlNb-based alloy. (**b**) Stereoscopic image showing coarse grains; (**c**) SEM back-scattered electron micrograph showing dark α_2_-Ti_3_Al, gray B + O-Ti_2_AlNb phases; (**d**) TEM bright field image along with (**e**–**g**) the corresponding SAED patterns of points 1 and 2, and crystallographic orientations, where 1 stands for O-Ti_2_AlNb phase, and 2 denotes B2 phase.

Ti_2_AlNb-based alloy contains different volumes of the ordered phases β_0_ (Strukturbericht: B2, space group: ${\rm{Pm}}\mathop{3}\limits^{-}{\rm{m}}$, Person symbol: cP2), α_2_-Ti_3_Al (Strukturbericht: DO_19_, space group: P6_3_/mmc, Pearson symbol: hP8), and the ordered orthorhombic O-Ti_2_AlNb phase (Strukturbericht: A_2_BC, space group: CmCm, Person symbol: oC16)^43^. There exist crystallographic orientations of these phases^1–3^: ${[1\bar{1}1]}_{B2}//{[11\bar{2}0]}_{{\alpha }_{2}}$, ${(011)}_{B2}//{(0001)}_{{\alpha }_{2}}$, ${[0001]}_{{\alpha }_{2}}//{[001]}_{O}$, ${(10\bar{1}0)}_{{\alpha }_{2}}//{(110)}_{O}$, ${[\bar{1}11]}_{B2}//{[1\bar{1}0]}_{O}$, ${(110)}_{B2}//{(001)}_{O}$. X-ray diffraction pattern, stereoscopic image, back-scattered electron (BSE) SEM micrograph, and TEM bright field image along with the relevant selected area electron diffraction (SAED) patterns of as-cast Ti_2_AlNb alloy are shown in Fig. 1(a–g). XRD results reveal the presence of O-Ti_2_AlNb phase, B2 phase, and α_2_ phase in Fig. 1(a). The stereoscopic image indicates coarse grains in the Ti_2_AlNb-cast alloy (Fig. 1(b)). The SEM image in Fig. 1(c) shows dark α_2_ phase, gray O-Ti_2_AlNb phase and B2 phase, which can be better seen in a magnified TEM image in Fig. 1(d). In Fig. 1(c), a small amount of α_2_ phase is mainly located at grain boundaries in the Ti_2_AlNb-based alloy. Then there are only B2 phase (matrix) and O-Ti_2_AlNb phase (lath) presented in Fig. 1(d–g), with crystallographic orientations between them: ${[\bar{1}11]}_{B2}//{[1\bar{1}0]}_{O}$, ${(110)}_{B2}//{(001)}_{O}$.

#### Isothermal oxidation kinetics

**Figure 2:**
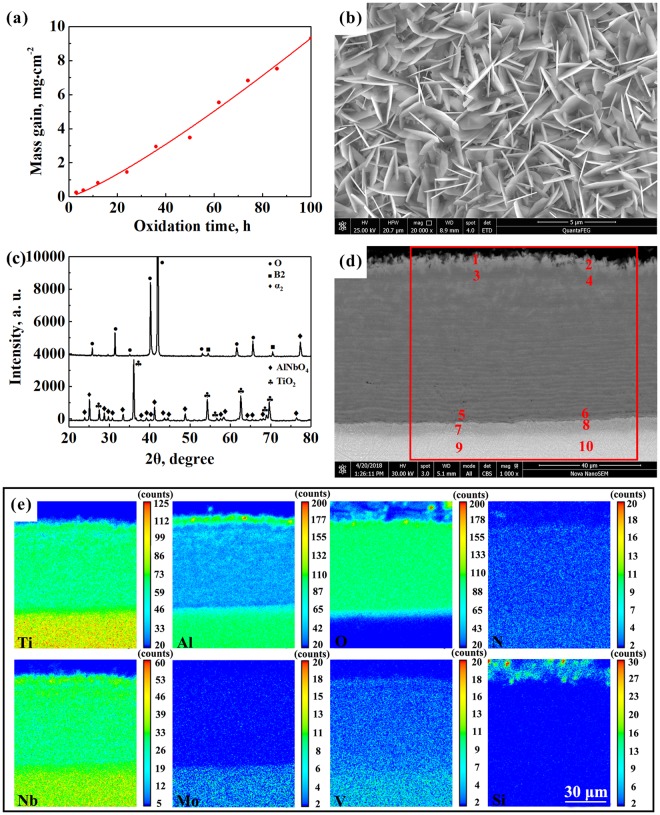
Oxidation of Ti_2_AlNb-cast alloy at 800 °C for 100 h. (**a**) Oxidation kinetics; (**b**) surface morphology of scale; (**c**) XRD pattern of the oxide scale on the surface and substrate; (**d**) SEM back-scattered electron micrograph of cross-structure scale; (**e**) EPMA mapping of a selected area in (**d**).

Figure 2(a) shows a curve of isothermal oxidation kinetics of Ti_2_AlNb-cast alloy at 800 °C. The obtained weight gain of this alloy after 100 h at 800 °C was about 9.3 mg/cm^2^. The relationship between oxidation and mass gain could be obtained by fitting the experimental data using the following equation,

1 $${\rm{\Delta }}{M}^{n}=k{}_{n}t,$$

where Δ*M* represents the weight gain per unit area (mg/cm^2^), *n* is an oxidation exponent (*n* = 1, liner relationship; *n* = 2, parabolic relationship), *k*_*n*_ is a rate constant (mg^*n*^/cm^2*n*^·h), and *t* is oxidation time (h). The obtained oxidation exponent was ~0.83, being close to 1, thus suggesting that the oxidation kinetics of Ti_2_AlNb-cast alloy at 800 °C obeyed basically a linear relationship and the oxide layer is not protective at this temperature.

### Surface morphology and structure of scale

Secondary electron micrograph, XRD pattern, back-scattered electron SEM micrograph, and EPMA mapping are shown in Fig. 2(b–e). Randomly-oriented and fairly-dense laminar-shaped oxides of about 2~3 μm long are observed to cover the alloy surface, as seen in Fig. 2(b).

X-ray diffraction patterns of oxide scale on Ti_2_AlNb alloy are shown in Fig. 2(c). It is seen that a large amount of AlNbO_4_ and TiO_2_ is present in the scale after oxidation at 800 °C. According to our previous studies^30^, the laminar-shaped oxide is AlNbO_4._ The cross-section of the overall scale can be seen from an SEM back-scattered electron (BSE) image shown in Fig. 2(d). The scale appears dense with a thickness of ~60 μm, and it consists of three layers based on the EPMA mapping in Fig. 2(e). The structure can be deduced to be (Al,Nb)-rich mixed oxide layer (I)/mixed oxide layer (II)/oxygen-rich layer (III) from the outside to inside. However, the presence of some Al_2_O_3_ is also found in layer I through TEM investigations.

EPMA point microanalyses were used to reveal the chemical composition in various locations of the cross-sectional scales in Fig. 2(d). According to XRD results in Fig. 2(c) and the chemical composition of points 1–10 in Table 1, AlNbO_4_ and TiO_2_ can be confirmed to be the main oxides in the scale. However, Al and Nb are richer at points 1–2 (in the outermost scale of ~5 μm thick) than at points 3–6, i.e., the (Al,Nb)-rich mixed outer layer (I) and mixed mid-layer (II). This should be a result of the rapid growth of TiO_2_. The consumption of oxygen leads to a reduction in the oxygen partial pressure, but there is still sufficient oxygen (compared to the interior of the alloy) in the O-rich zone (III), as represented by points 7–10 where the content of oxygen was determined to be ~15 at.% or higher, as shown in Table 1. Also, a low content of nitrogen indicates that there is no nitride present in the oxide scale (points 1–6). N and O penetrated into O-rich zone (III) will cause an environmental brittleness and reduce the mechanical properties of the alloy^28^.

**Table 1:** Elemental composition (at.%) determined via EPMA at points 1–10 in Fig. 2(d).

Number	Ti	Al	Nb	Mo	V	Si	O	N
1	11.88	12.03	11.64	0.00	0.93	0.03	63.50	0.00
2	12.75	11.04	12.26	0.00	0.96	0.03	62.96	0.00
3	22.56	5.63	6.82	0.00	0.61	0.03	62.92	1.43
4	23.41	5.22	6.59	0.00	0.64	0.05	62.66	1.43
5	23.40	5.52	6.86	0.00	0.97	0.07	61.73	
6	21.28	6.63	7.72	0.00	0.92	0.11	62.15	1.19
7	34.09	12.01	13.28	0.20	1.38	0.16	34.35	4.52
8	36.66	12.59	14.15	0.24	1.58	0.21	29.07	5.51
9	43.29	16.02	14.84	0.22	1.67	0.20	17.54	6.23
10	44.93	15.92	16.85	0.26	1.82	0.18	14.51	5.54

#### TEM study of oxide layer

**Figure 3:**
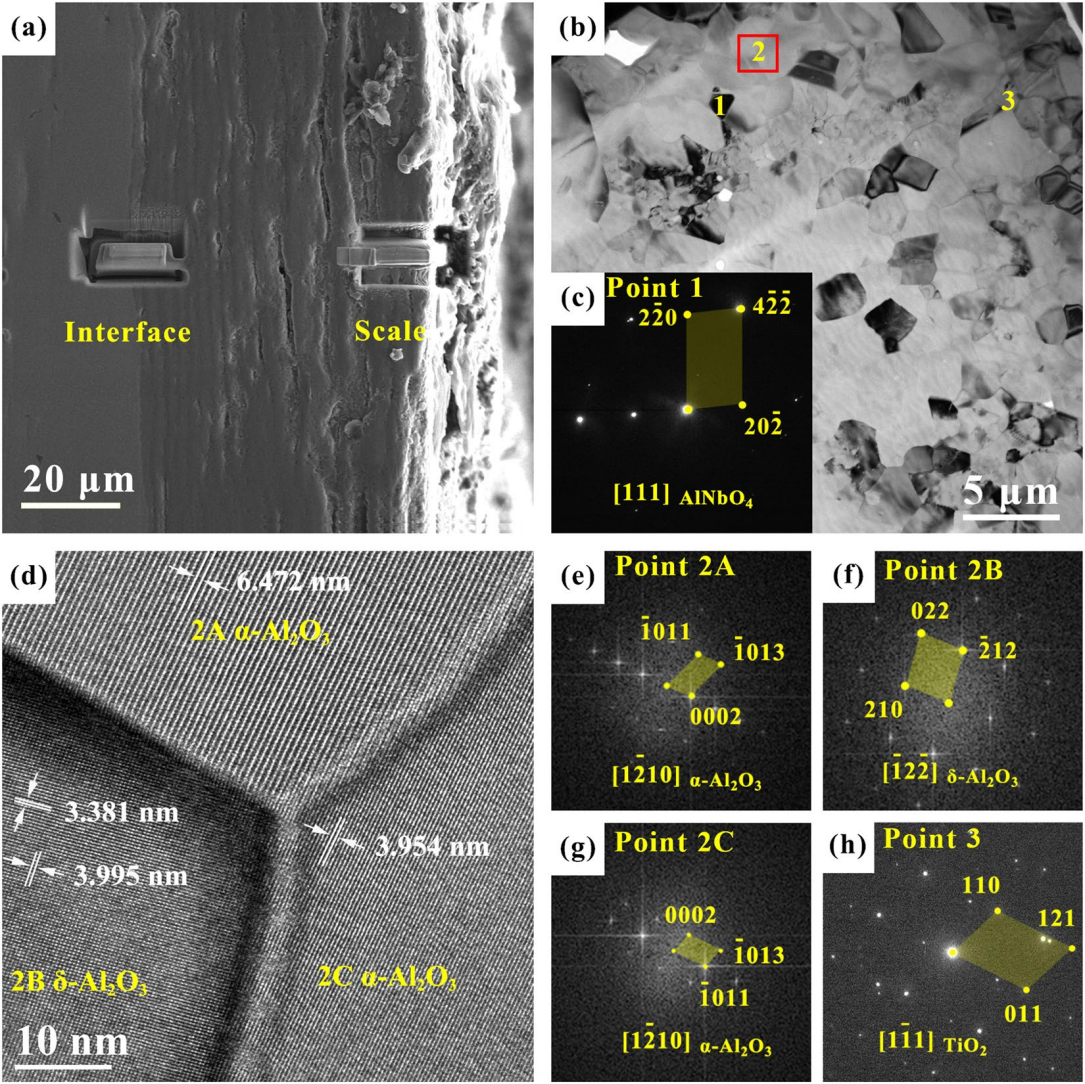
Analysis of oxide scale after oxidation at 800 °C for 100 h. (**a**) Position of TEM samples prepared via FIB; (**b**) TEM bright field image; (**c**) and (**h**) the corresponding SAED patterns of point 1 and 3 in (**b**), and (**d**–**g**) the corresponding HRTEM and FFT images of point 2 in (**b**), where 1 represents AlNbO_4_, 2 A denotes α-Al_2_O_3_, 2B stands for δ-Al_2_O_3_, 2 C signifies α-Al_2_O_3_, and 3 indicates rutile-TiO_2_.

TEM samples were taken from the outer scale (right) and at the scale/substrate interface (left) via FIB, as shown in Fig. 3(a). Figure 3(b) shows a TEM bright-field image, with the SAED pattern of point 1 given in Fig. 3(c), indicating the presence of AlNbO_4_ phase. Figure 3(d) is an HRTEM image of point 2 in Fig. 3(b), where Fourier transformations of 2A, 2B, and 2 C were performed to obtain diffraction patterns shown in Fig. 3(e) to (g), corresponding to α-Al_2_O_3_, δ-Al_2_O_3_ and α-Al_2_O_3_. This suggests the co-existence of different types of Al_2_O_3_ oxides. It should be noted that, to the best of the authors’ knowledge, such a co-existent phenomenon of different forms of Al_2_O_3_ oxides observed via HRTEM has not been reported in the literature, where α-Al_2_O_3_ is a thermodynamically stable form (corundum form) while δ-Al_2_O_3_ is one of metastable transition forms/polymorphs of alumina. Figure 3(h) is a SAED pattern of point 3 in Fig. 3(b), which corroborates the presence of TiO_2_.

**Figure 4:**
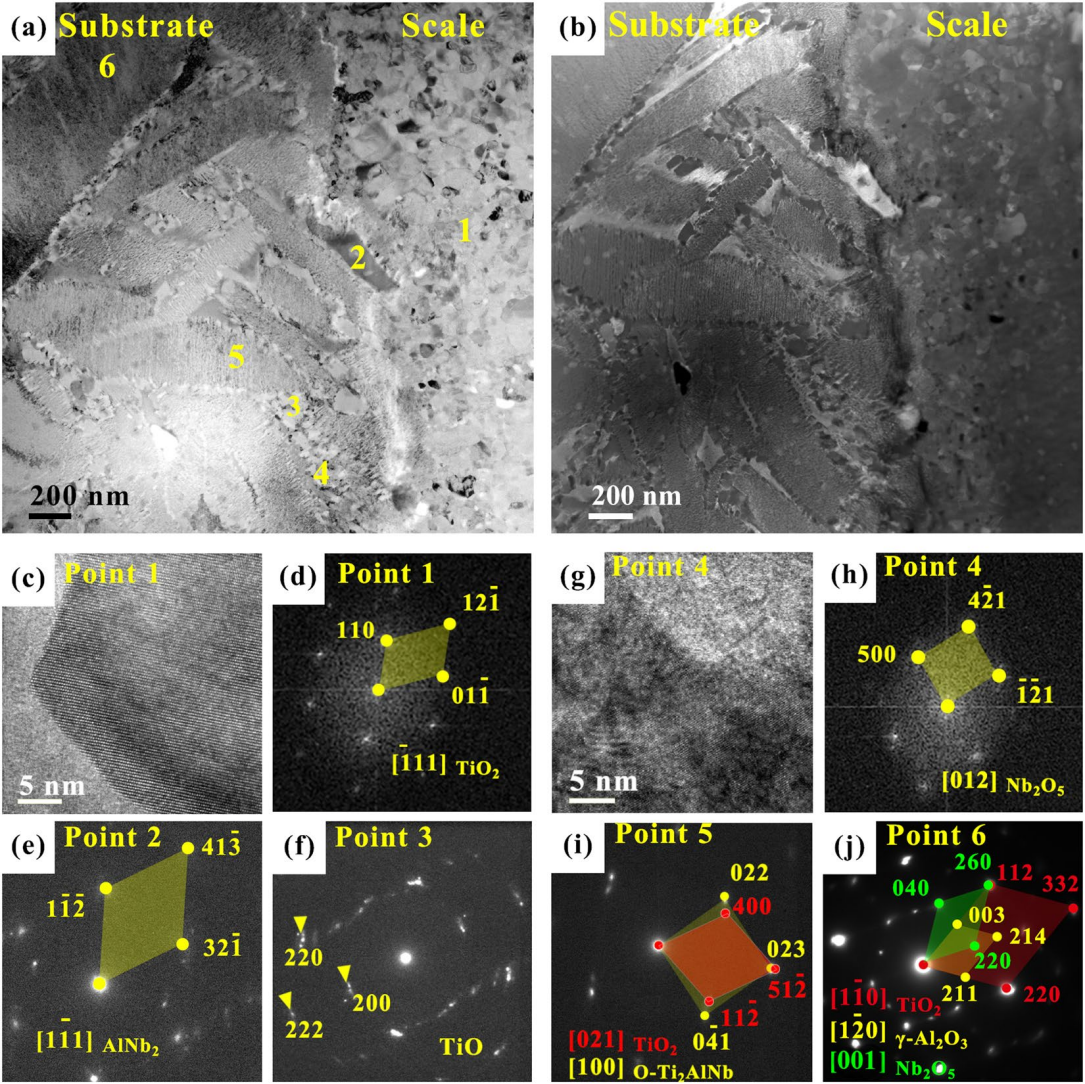
Microstructures of interface between as-cast Ti_2_AlNb-based alloy substrate and oxide scale. (**a**) TEM bright field image of interface, where white regions reflect pores, (**b**) high angle annular dark field (HAADF) image of (**a**); (**c**) HRTEM of point 1, and (**d**) FFT image of (**c**); (**g**) HRTEM of point 4, and (**h**) FFT image of (**g**); (**e**), (**f**), (**i**) and (**j**) the corresponding SAED patterns of points 2, 3, 5, 6, where point 1 represents rutile-TiO_2_, point 2 represents AlNb_2_, point 3 represents TiO, point 4 represents Nb_2_O_5_, point 5 represents brookite TiO_2_ and O-Ti_2_AlNb phase, point 6 represents rutile TiO_2_, Nb_2_O_5_ and γ-Al_2_O_3_.

Figure 4 shows the microstructures of interface between Ti_2_AlNb substrate and oxide scale, from a TEM sample taken at the interface of oxide layer/substrate (i.e., the left TEM sample shown in Fig. 3(a)). Figure 4(a,b) present a TEM bright-field image and a high angle annular dark-field (HAADF) image, respectively, where the left side represents the substrate and the right side represents the oxide scale. It should be noted that the white spots in the oxide layer in Fig. 4(a) are pores. The rutile-TiO_2_ is found at point 1 in Fig. 4(c,d), with an amorphous surrounding. Nb content at point 2 is very high, and it has been identified as AlNb_2_ (Fig. 4(e)), which is also one of the common oxidation products of the Ti_2_AlNb-based alloy. Point 3 shows a TiO polycrystalline ring (Fig. 4(f)), which is further oxidized to become TiO_2_. In Fig. 4(g,h) for point 4, Nb_2_O_5_ is revealed to be present at the boundary of two phases. This is due to the fact that the diffusion of oxygen at the phase boundary is faster as a result of the presence of phase (or grain) boundary energy, along with the reduced reaction activity of Ti and Al. Point 5 shows a lamellar O-Ti_2_AlNb phase where brookite TiO_2_ and O-Ti_2_AlNb phases are present, which are shown in Fig. 4(i). In Fig. 4(j), TiO_2_, γ-Al_2_O_3_, and Nb_2_O_5_ are observed to co-exist in the O-Ti_2_AlNb laths, with the orientation relationships of γ-Al_2_O_3_ and TiO_2_: ${(211)}_{\gamma -A{l}_{2}{O}_{3}}//{(220)}_{rutile-Ti{O}_{2}}$, ${(003)}_{\gamma -A{l}_{2}{O}_{3}}//{(112)}_{rutile-Ti{O}_{2}}$, $\,{[1\bar{2}0]}_{\gamma -A{l}_{2}{O}_{3}}//{[1\bar{1}0]}_{rutile-Ti{O}_{2}}$.

## Discussion

### Evolution of B2 phase and O-Ti_2_AlNb phase

As mentioned above, the Ti_2_AlNb-based alloy consists of 3 phases, i.e., α_2_-Ti_3_Al, B2, and O-Ti_2_AlNb. In the present Ti_2_AlNb-cast alloy there is fewer α_2_-Ti_3_Al phase existing in the vicinity of grain boundaries and the O-Ti_2_AlNb laths are present in the original coarsened B2 grains. The diffusion rate of oxygen atoms in the B2 phase is larger than that in the other two phases^44^. It follows that no B2 phase is present at the interface. This means that oxidation reaction occurs first in the B2 phase, producing metastable TiO, which is further oxidized to TiO_2_. The brightest regions on the HAADF image in Fig. 4(b) are Nb-rich areas and have been identified to be AlNb_2_. It is sandwiched between the complete and residual O-Ti_2_AlNb laths, which corresponds to the former position of the B2 phase. AlNb_2_ is considered as a by-product during the oxidation of the Nb-containing TiAl-based alloy, which is due to the reaction of enriched Nb and Al elements^45^. There are a large number of Nb element in the B2 phase^43^. During oxidation, the Ti element in the B2 phase reacts with the O element to form TiO/TiO_2_. The consumption of Ti causes the reaction of Al and Nb. As a result, AlNb_2_ is surrounded by gray TiO/TiO_2_ in Fig. 4(b). AlNb_2_ is stably present at the interface, indicating that its oxidation resistance is relatively strong^45^, however, the discontinuous nature of AlNb_2_ at the interface makes the effect limited.

After this the O-Ti_2_AlNb phase is oxidized. Combined with Fig. 4(f–j), there are brookite-TiO_2_, rutile-TiO_2_, γ-Al_2_O_3_, and Nb_2_O_5_ in the O-Ti_2_AlNb phase. It can thus be considered that the O-Ti_2_AlNb phase has been oxidized and decomposed into oxides of TiO_2_, Al_2_O_3_, and Nb_2_O_5_. Al_2_O_3_ blocks the transport channel, hinders further growth of TiO_2_, and improves oxidation resistance. As one of the oxide products, Al_2_O_3_ has been reported extensively in the literature. During the early stage of oxidation, the oxides at the interface exist in an amorphous form. Lu *et al*.^29^ reported that polycrystalline TiO_2_ and amorphous Al_2_O_3_ coexist in the scale. This is similar to the structure in Fig. 4(c). It should be noted that TiO_2_ and Al_2_O_3_ occur almost simultaneously because of their similar Gibbs free energy^43^, however, the energy is not high enough at an oxidation temperature of 800 °C, resulting in the presence of metastable alumina. To the best of the authors’ knowledge, there is no report about the structure of Al_2_O_3_ in the oxidation of a Ti_2_AlNb-based alloy. In the present work, the unique formation of alumina is observed via HRTEM in the alloy after oxidation at 800 °C for 100 h. That is, the presence of three adjoining oxide grains and oxides in O-Ti_2_AlNb laths, as shown in Figs 3(d) and 4(j), suggests the occurrence of phase change among three types of Al_2_O_3_, i.e., α-Al_2_O_3_, δ-Al_2_O_3,_ and γ-Al_2_O_3_. The γ-Al_2_O_3_, θ-Al_2_O_3_, and κ-Al_2_O_3_ are common metastable phases in TiAl alloys at 900 °C^29,46,47^. According to Yang *et al*.^48^, γ-Al_2_O_3_ formed with twins in the oxidation of NiAl, and γ-Al_2_O_3_ twins were observed to play an important role in the scale growth. Cowley *et al*.^49^ reported that {111} γ-Al_2_O_3_ twin boundaries provide a fast diffusion path for Al cations. This would improve the oxidation resistance of an alloy. However, in the present study of the Ti_2_AlNb-based alloy at 800 °C, there exists δ-Al_2_O_3_ (monoclinic, *a* = 11.74 Å, *b* = 5.72 Å, *c* = 11.24 Å, *β* = 103.34°^50^), while no γ-Al_2_O_3_ twins are observed. As reported by Levin and Brandon^51^, there is a route for the formation of Al_2_O_3_: Amorphous (anodic film) → γ → δ → θ → α-Al_2_O_3_. This would be the phase change route of Al_2_O_3_ in the present Ti_2_AlNb-based alloy. Further studies in this aspect are needed at different oxidation temperatures.

### Formation of AlNbO_4_

**Figure 5:**
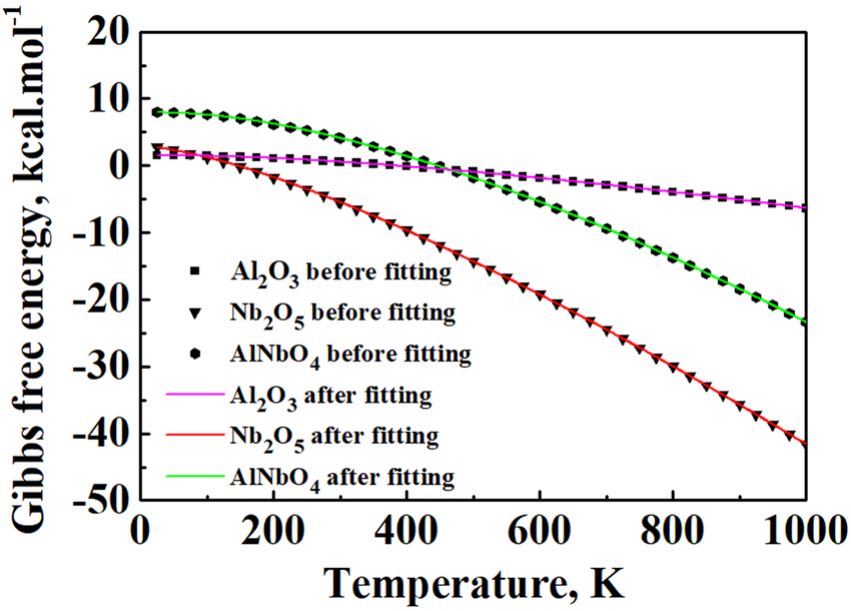
Gibbs free energy curves of Al_2_O_3_, Nb_2_O_5_ and AlNbO_4_ before and after fitting.

As described above, there are TiO_2_, Al_2_O_3_, and AlNbO_4_ in the outer oxide layer, as presented in Fig. 3. Zheng *et al*.^40^ assumed that the fast-growing Nb_2_O_5_ could react with Al_2_O_3_ that developed at the early stage of oxidation to form AlNbO_4_. In the present work, Dmol3 module in Materials Studio 6.0 is used to calculate the Gibbs free energy change of reaction: Nb_2_O_5_ + Al_2_O_3_ → 2AlNbO_4_, however, the temperature in the software is up to 1000 K only, which is lower than the temperature of the present oxidation experiment, 1073.15 K. Thus, the following equation is used to fit the relationship between the Gibbs free energy and temperature,

2 $$G={H}_{0}+aT\,\mathrm{ln}\,T+b{T}^{2}+\frac{c}{T}+IT,$$

where *H*_0_, *a*, *b*, *c*, and *I* are fitting parameters. The results show an excellent matching close to 100%, as shown in Fig. 5 and Table 2. The relationships between the Gibbs free energy and temperature for Al_2_O_3_, Nb_2_O_5_, and AlNbO_4_ can thus be expressed as follows,

3 $${{\rm{G}}}_{{{\rm{Al}}}_{2}{{\rm{O}}}_{3}}=1.18577-0.00368T\,\mathrm{ln}\,T-1.4271\times {10}^{-6}{T}^{2}+\frac{3.88868}{T}+0.01932T,$$

4 $${{\rm{G}}}_{{{\rm{Nb}}}_{2}{{\rm{O}}}_{5}}=2.07990-0.01388T\,\mathrm{ln}\,T-2.54067\times {10}^{-6}{T}^{2}+\frac{10.8132}{T}+0.05475T,$$

5 $${{\rm{G}}}_{{{\rm{AlNbO}}}_{4}}=6.74954-0.01287T\,\mathrm{ln}\,T-8.12388\times {10}^{-6}{T}^{2}+\frac{16.08122}{T}+0.06692T.$$

**Table 2:** Fitting results of free energy curve of Al_2_O_3_, Nb_2_O_5_ and AlNbO_4_ in Fig. 5.

Phase	*H*_0_	*a*	*b*	*c*	*I*	Adj. R-Square
Al_2_O_3_	1.18577	−0.00368	−1.4271E-6	3.88868	0.01932	1
Nb_2_O_5_	2.0799	−0.01388	−2.54067E-6	10.8132	0.05475	1
AlNbO_4_	6.74954	−0.01287	−8.12388E-6	16.08122	0.06692	0.99999

Substituting *T* = 1073.15 K into the above equations yields the Gibbs free energy values for the formation of Al_2_O_3_, Nb_2_O_5_, and AlNbO_4_, respectively, in the present oxidation condition of a higher temperature. As a result, the Gibbs free energy change of the reaction Nb_2_O_5_ + Al_2_O_3_ → 2AlNbO_4_ becomes −1.00873 kJ/mol. This means that the reaction is thermodynamically possible. Also, Ai *et al*.^52^ reported that Nb_2_O_5_ reacted completely with Al_2_O_3_ to form AlNbO_4_ in Nb_2_O_5_-Al_2_O_3_ ceramics. While Nb_2_O_5_ is consumed as a reactant, the formed AlNbO_4_ makes the scale unprotective as well^14^. Furthermore, Nb_2_O_5_ is not observed in the XRD results (Fig. 2(c)), which can be mainly attributed to its reaction with Al_2_O_3_ as discussed above, in conjunction with the lower diffusion coefficient of Nb, the rapid growth of TiO_2_, and the hindering effect of alumina.

### Oxidation process at the interface

**Figure 6:**
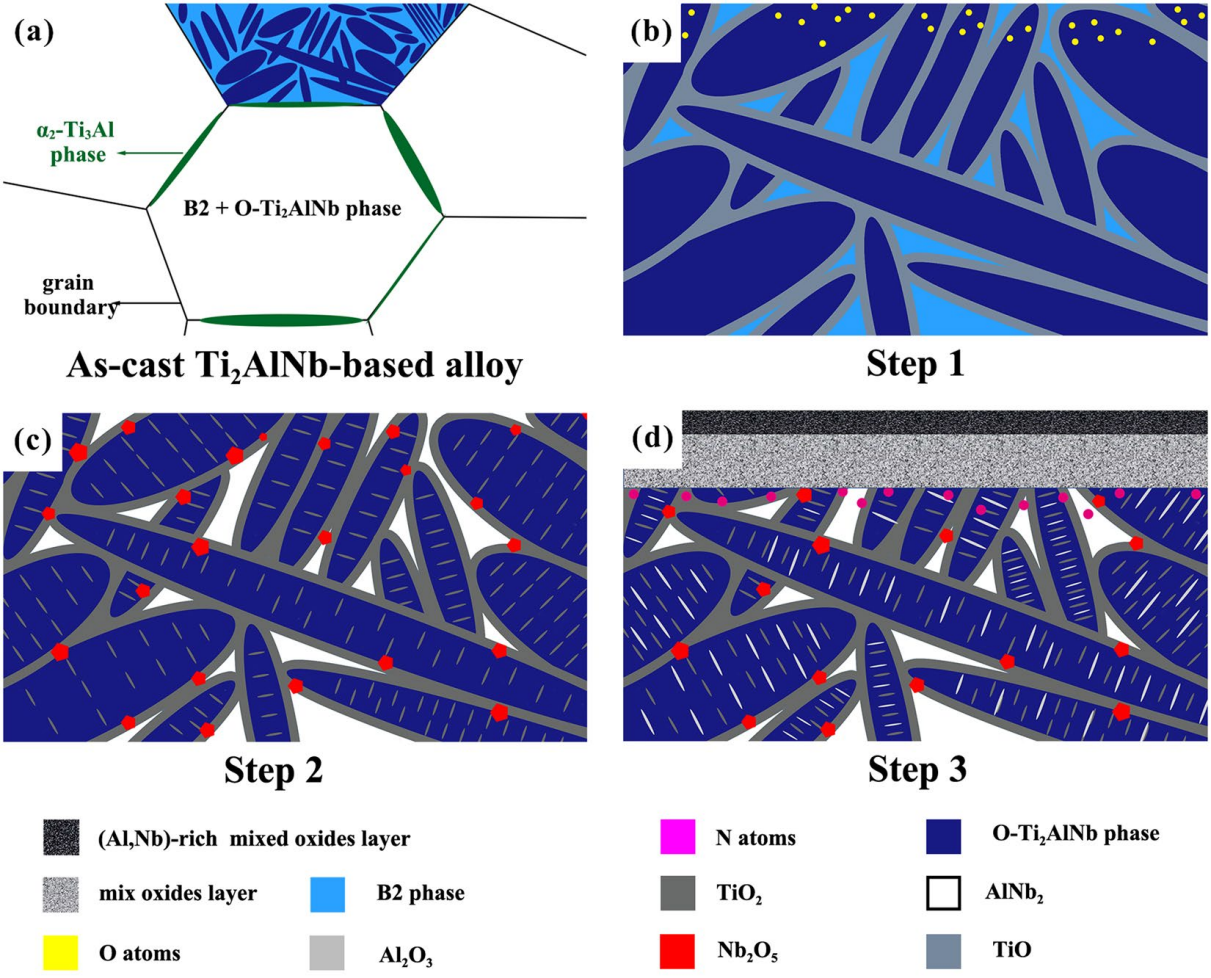
Schematic diagram showing a summary of high-temperature oxidation process of Ti_2_AlNb-based alloy. Stage 1, by inward diffusion of oxygen B2 phase is transformed into TiO, and Ο-Ti_2_AlNb is abound with oxygen. Stage 2, TiO is transformed into rutile-TiO_2_ and AlNb_2_ is formed in the areas of B2 phase. Oxidation occurs in O-Ti_2_AlNb phase. Stage 3, O-Ti_2_AlNb phase breaks down into Al_2_O_3_, Nb_2_O_5_ and TiO_2_. Al_2_O_3_ reacts with Nb_2_O_5_ to form (AlNbO_4_ + TiO_2_) mixed oxide layer. At last, nitrogen dissolves in the alloy.

Based on the above observations and analyses, the high-temperature oxidation process of the Ti_2_AlNb-based alloy can be summarized below and schematically shown in Fig. 6. Stage 1: Oxygen absorbs on the surface of the Ti_2_AlNb-based alloy, which later penetrates into it. The B2 phase is oxidized to produce TiO, and oxygen dissolves in O-Ti_2_AlNb phase. Stage 2: TiO is transformed into rutile-TiO_2_ and AlNb_2_ is formed in the areas of the B2 phase. Oxidation occurs, i.e., brookite-TiO_2_ is generated inside the O-Ti_2_AlNb phase and Nb_2_O_5_ outside the O phase. Stage 3: O-Ti_2_AlNb phase breaks down to TiO_2_, Nb_2_O_5_, and Al_2_O_3_. In the scale there is a reaction: Al_2_O_3_ + Nb_2_O_5_ → 2AlNbO_4_, and N atoms are dissolved in the alloy because of the consumption of O atoms. The corresponding crystallographic structures of the related oxides of the Ti_2_AlNb-based alloy are summarized in Table 3.

**Table 3:** Crystallographic structure of the related oxides.

Phase	PDF number	Space group	Crystal system
AlNbO_4_	#41-0347	C2/m (12)	Monoclinic
γ-Al_2_O_3_	#10-0425	Fd-3m (227)	Cubic
δ-Al_2_O_3_	#11-0517	C2/m (12)	Monoclinic
α-Al_2_O_3_	#46-1212	R-3c (167)	Hexagonal
Rutile-TiO_2_	#21-1276	P42/mnm (136)	Tetragonal
Brookite-TiO_2_	#29-1360	Pcab (61)	Orthorhombic
Nb_2_O_5_	#22-1196	A2/m (12)	Monoclinic
	#19-0864	Primitive	Monoclinic
AlNb_2_	#15-0598	P42/mnm (136)	Tetragonal

## Conclusions

1. After being exposed at 800 °C in static air for 100 h, the Ti_2_AlNb-based alloy followed an almost liner kinetic law of oxidation and exhibited a multi-layered structure consisting of an (Al,Nb)-rich mixed oxide layer (I), mixed oxide layer (II), and oxygen-rich layer (III) from the outside to inside.
2. In the mixed outer scale, there existed α-Al_2_O_3_ and δ-Al_2_O_3_. Al_2_O_3_ reacted with Nb_2_O_5_ to form AlNbO_4_, however, Nb_2_O_5_ and AlNbO_4_ were not able to hinder the diffusion of oxygen.
3. The B2 phase was oxidized to form TiO_2_, where Nb and Al were transformed into AlNb_2_ at the interface during oxidation. AlNb_2_ could hinder the diffusion of oxygen and improve the oxidation resistance of the Ti_2_AlNb-based alloy, but its discontinuous nature allowed only a limited effect.
4. After long-term oxidation at 800 °C, O-Ti_2_AlNb was oxidized to form TiO_2_, Al_2_O_3_ and Nb_2_O_5_. Al_2_O_3_ could hinder the growth of TiO_2_ in the O-Ti_2_AlNb laths and form a compact scale. Increasing the amount of the O-Ti_2_AlNb phase in the alloy contributes to the improvement in its high-temperature oxidation resistance.

## Data Availability

The datasets generated and/or analyzed during the current study are available from the corresponding author on reasonable request.
